# Energy and thermal modelling of an office building to develop an artificial neural networks model

**DOI:** 10.1038/s41598-022-12924-9

**Published:** 2022-05-27

**Authors:** Jose Maria Santos-Herrero, Jose Manuel Lopez-Guede, Ivan Flores Abascal, Ekaitz Zulueta

**Affiliations:** 1 Department of Energy Engineering, Faculty of Engineering in Bilbao, University of the Basque Country (UPV/EHU), Plaza Torres Quevedo 1, 48013 Bilbao, Spain; 2 Systems and Automatic Control Department, University Faculty of Engineering of Vitoria, University of the Basque Country (UPV/EHU), c/Nieves Cano 12, 01006 Vitoria-Gasteiz, Spain; 3 Department of Energy Engineering, Faculty of Engineering in Bilbao, University of the Basque Country (UPV/EHU)/ENEDI Research Group, Plaza Torres Quevedo 1, 48013 Bilbao, Spain

**Keywords:** Engineering, Civil engineering, Electrical and electronic engineering, Energy infrastructure, Energy infrastructure

## Abstract

Nowadays everyone should be aware of the importance of reducing CO_2_ emissions which produce the greenhouse effect. In the field of construction, several options are proposed to reach nearly-Zero Energy Building (nZEB) standards. Obviously, before undertaking a modification in any part of a building focused on improving the energy performance, it is generally better to carry out simulations to evaluate its effectiveness. Using Artificial Neural Networks (ANNs) allows a digital twin of the building to be obtained for specific characteristics without using very expensive software. This can simulate the effect of a single or combined intervention on a particular floor or an event on the remaining floors. In this paper, an example has been developed based on ANN. The results show a reasonable correlation between the real data of the Operative Temperature with the Energy Consumption and their estimates obtained through an ANN model, trained using an hourly basis, on each of the floors of an office building. This model confirms it is possible to obtain simulations in existing public buildings with an acceptable degree of precision and without laborious modelling, which would make it easier to achieve the nZEB target, especially in existing public office buildings.

## Introduction

In the European construction sector, the nearly-Zero Energy Buildings (nZEB) regulation has been issued in the Energy Performance of Buildings Directive (EPDB) 2010/31/EU^1^ for all new buildings after 31 December 2020. However, the large number of existing buildings that still do not comply with these standards, as well as their slow renovation process, as indicated in the research developed by D'Agostino et al.^2^ and Ruparathna et al.^3^, make it necessary to propose alternatives in order to be able to achieve these CO_2_ emission reduction targets through other solutions. In the case of existing buildings, the most common renovation strategies are usually focused on envelope improvement (for instance, window replacement as suggested by Aste et al.^4^, envelope insulation improvement as indicated by Terés-Zubiaga et al.^5^ and even the development of new materials as proposed by Chung et al.^6^). Other strategies are based on the renovation of Heating, Ventilation and Air Conditioning (HVAC) systems; either by improving existing equipment as proposed by Che et al.^7^ and Dascalaki et al.^8^, or by integrating systems based on renewable energy sources as developed by Caskey et al.^9^ and Dipasquale et al.^10^.

In order to explore other alternatives, the use of work philosophies applied to the industrial field has been considered, where the search for the optimization of their processes has continued for years in order to achieve maximum efficiency, as suggested by Drgoňa et al.^11^. To fulfil this target, the Building Energy Performance Simulation (BEPS) tools are an interesting option and they could be of great help in finding solutions that allow energy consumption to be optimized with a relatively low economic investment. Nevertheless, these developments require a great effort, given the enormous amount of data that is collected in the field; namely environmental conditions (temperatures and humidity) and energy consumption. Furthermore, it is usual to carry out a calibration process to debug the information for the simulation data, so as to have a correlation to the real information captured in the building with the minimum possible margin of error. In addition, considering that the users of the building can also cause disturbances, as indicated by Oldewurtel et al.^12^; these tasks are laborious and require specific knowledge, a great amount of time, and an economic cost through licenses for computer programs and equipment, which could be an important handicap.

Therefore, in this research, we have proposed to simplify and reduce the economic cost of the process. In order to do this, we have developed an algorithm based on Artificial Neural Networks (ANN) that allows us to obtain the Operative Temperatures and Energy Consumption of the different areas of an office building, in our case the Rectorate building of the University of the Basque Country located in northern Spain.

This paper explains the research developed, dividing it into 5 sections: Sect. 1 explains the motivations and gives a global vision of the study, as well as a short review of the different current BEPS tools which allow energy modelling and simulations of any type of building. Section 2 sets out the background of the methodology applied and gives a brief review of the research that justifies this work. Section 3 provides a case study of how the synthetic data used has been generated through modelling versus the real data from 2016 to 2019; the developed ANN algorithm is also presented. In Sect. 4, the results are justified by considering that the margins of error achieved are acceptable. Finally, the conclusions are summarized in Sect. 5.

### Literature review

Current BEPS tools allow energy modelling and simulations of any type of building. Several investigations have been carried out successfully in recent years applying this type of BEPS software. Among the various BEPS tools are: *DesignBuilder*, *IDA-ICE*, *EnergyPlus*, *CPLEX*, *TRNSYS*, *DOE-2* or *ESP-r*, which allow the given energy consumption to be minimized and the most appropriate strategies respecting the established comfort requirements to be identified.

In this sub-Section, various investigations^13–29^ are indicated that justify this. For example, Murano et al.^13^ applied *DesignBuilder* to explain the imbalance between heating and cooling in the different climatic zones of Italy in different types of buildings; while Cornaro et al.^14,15^ characterized several commercial elements: a Phase Change Materials (PCM) panel, which was validated with a PCM computerized tool using *IDA-ICE* as well as a benchmarking of potential energy savings analysis among four types of “Photovoltaic Semi-Transparent Materials” with respect to conventional double panel glass. In addition, the *IDA-ICE* tool was used by Kurnitski et al.^16^ to estimate nZEB energy efficiency levels in compliance with “Federation of European Heating, Ventilation and Air-conditioning Associations” requirements, along with its optimal cost. Other papers have developed their research applying *EnergyPlus*; for instance Loukaidou et al.^17^ carried out experiments with several test-cell buildings for cost-optimal analysis according to the characteristics of the building envelope; Kang^18^ performed some research to optimize the economic sustainability of the “Life Cycle Cost” with the aim of applying energy saving methods from the early phases in the design of a building; Becchio et al.^19^ presented a roadmap to establish net zero energy balance measures with high energy efficient technical systems to optimize the insulation of the envelope, which was tested on a single family house nZEB in Northern Italy; Barthelmes et al.^20^ explained an example of a newly built single-family house applying a cost-optimal methodology from a preliminary design phase of the project; Adhikari et al.^21^ proved that a zero energy building can have a viable investment cost, especially if Photovoltaic (PV) facilities are used. Furthermore, the *CPLEX* tool was also used in the case of González et al.^22^, who assessed an optimization model to evaluate on-site RES composed by mini-wind turbines, solar PVs and a battery storage which was tested in a real building in Portugal to minimize its annual energy costs.

However, one of the BEPS tools most applied, with the best results and an acceptable accuracy, has been *TRNSYS*. Aste et al.^23^ reviewed the energy-efficiency of an integrated multifunctional system to provide the HVAC demands using RES consisting of PVs, aerothermal energy and Heat Pumps (HP) on residential dwellings; Bozkaya et al.^24^ explored a dynamic co-simulation method with three different insulation parameters to look into the influence of the thermal imbalance of aquifer thermal energy storage systems and building thermal load; Ogando et al.^25^ developed a real case of the application of energy modelling and deterministic calibrations in an elementary school in Galicia (Northwest Spain); Baglivo et al.^26^ modelled several design options and different configurations of a construction prototype situated in a warm weather climate to compare its thermal behaviour and optimize the design of its envelope; Peán et al.^27^ analysed the impact on a demand-side energy management control strategy by simulating a refurbished dwelling nZEB; Palme et al.^28^ presented a new simulation methodology considering the effects of the urban microclimate through a climate model, the “Urban Weather Generator”; while Iturriaga et al.^29^ carried out a simulation work with a “Mixed-Integer Linear Programming” model, which was implemented in an existing building in the Basque Country (Northern Spain), where energy saving actions were adopted to optimize the power requirement in the retrofitting of existing buildings in order to reach the nZEB standard.

## Methodology

### Background of the methodology

The “*Kaizen*” philosophy is the most widespread in advanced companies in the industrial sector. In this philosophy, continuous improvement is a fundamental pillar, in which the origin of possible inefficiencies must be analysed under the “*Muda*” concept: “*The 7 big wastes*”. Each of these typical wastes in the industrial field have a correlation in building climatization, if it is to be considered as an industrial process:

- **Defects** … any building climatization has energy losses. It is very important to take care with the building’s degree of infiltration, avoiding losses for bad insulation of the piping net or other devices. A previous analysis and design phase can largely prevent certain types of defect or minimize the risk that they may happen.
- **Transport** … obviously, if the energy is produced at a great distance from its point of consumption, losses will occur during its transport. It would be very interesting, whenever possible, to generate the energy at its point of consumption through the use of solar with water accumulators and / or photovoltaic panels on the roof.
- **Time** … this concept can be interpreted in building climatization as taking into consideration the thermal inertia of the building itself and the importance of knowing in advance how much energy is required at any time.
- **Overproduction** … it would be interesting to avoid hot water immobilized for hours waiting for its demand. Applying the "*Just In Time*" concept in the building climatization would lead to the production of hot water only and exclusively when it is required.
- **Inventory** … the self-stored energy as the thermal inertia of the building itself, which can have a positive or negative contribution, depending on how it is managed.
- **Movements** … wasting energy without a clear target does not make sense either. That is why it is important to have a roadmap established where what we want to do at all times is planned, depending on the external conditions as well as the existing needs at any given time.
- **Processes** … depending on the way a production and air conditioning process is defined, it will be more or less efficient. It would be very interesting to establish an equation or algorithm that takes into account all the wastes that can be generated in the building climatization so as to minimize them as much as possible.

For this purpose, it has been proposed to apply modelling and simulation tools focusing on the last 5 wastes indicated, given that they will facilitate the tasks of:

- Previously knowing the amount of energy required at each moment,
- Planning energy needs at all times based on external conditions and comfort requirements,
- Reaching an algorithm that allows all the data needed to minimize the energy consumption to be managed.

### Justification on this research

Considering the various works of research carried out, some of which are mentioned in the subsection "1.2. Literature review", the modelling and simulation process of a building is a laborious process that requires costly technical and human resources. Moreover, this process usually requires a calibration with real measurements to make a final adjustment to the simulation. In this situation, where it is necessary to collect field data, recording real information to adjust the model and make its simulation closer to reality, it has led us to propose the development of a new working hypothesis. The said hypothesis is to design an ANN model based on real measurements, defined as inputs, that represent the main factors which have an impact on the air conditioning system of a building; and also to validate its response with the outputs that have been considered: the Operative Temperatures and the Energy Consumption. All these data were taken on an hourly basis, for each of the floors of a public office building.

This approach through an ANN model could significantly reduce the necessary resources, since it would not be necessary to develop an entire modelling process with the time involved in collecting drawings, documentation and information on the building envelope. Neither would a full simulation process be required, with its subsequent calibration, because the database of real measurements is used as the basis for developing the ANN model. Therefore, if reliable estimates of the Operative Temperature and Energy Consumption of a public building could be made through an ANN model, this would open up the possibility of reducing the modelling and simulation process of any building. This is our working hypothesis, starting from real data captured in the building itself, without modelling and the subsequent calibration process, aiming to emulate the energy and thermal behaviour of the building under study with reasonable accuracy.

Nowadays, the accuracy of the BEPS tools and their calibration processes are checked with the criteria set out by such different global organizations as the Federal Energy Management Program (FEMP), with its Measurement and Verification guidelines (M&V)^30^, the International Performance Measurement and Verification Protocol (IPMVP)^31^, or the Guideline 14 of the American Society of Heating, Ventilating, and Air Conditioning Engineers (ASHRAE)^32^. All of them apply some standardized statistical indices, the Mean Bias Error (MBE) (%) or the Coefficient of Variation of Root Mean Square Error CV(RMSE) (%), where the Root Mean Square Error (RMSE) is applied to assess the performance of a calibration or the simulation model. For instance, by applying an hourly basis, ASHRAE and FEMP set the maximum percentage diversion at ± 10%, while IPMVP sets it at ± 5%. This can establish whether a calibration can be considered as acceptable. However, it cannot be guaranteed that the input data applied in the BEPS are in line with reality. In our research, the work carried out by Martin-Escudero et al.^33^, on a part of the same building as in our study, has been taken as the calibration reference. There, the West block was monitored to check its energy behaviour by simulating it for a year with the *EnergyPlus* simulation tool. In that research, the result of the calibration assessment (obtained in one winter week with an hourly basis) for the heating energy consumption was MBE = -3.70%.

Accordingly, with the current Machine Learning models, it is possible to carry out predictive analytics on any type of data, especially in its application to Sustainable Energy Systems, as extensively explained by Donti et al.^34^ using algorithms that imitate human cognitive functions through ANN because they stand out in the analysis and optimization processes. Therefore, there is a great potential in using these tools to optimize the methodology applied in the modelling and simulation of the thermal behaviour, as well as the energy consumption of a building, as indicated by Santos-Herrero et al.^35^ in its overview of MPC in building climatization.

In addition, for the reasons given at the end of the subsection "1.2. Literature review", the *TRNSYS* software has been used, as it is considered a sufficiently validated tool. This transient systems simulation program was developed by the University of Wisconsin (USA) in 2010. It has been improved with different updates, making it a BEPS tool that allows the characteristics of the envelope, the conditions of use and the thermal behaviour of any building to be simulated. For our research, the *TRNSYS* version 17^36^ has been used to generate the synthetic data, which have allowed us to generate a modelling and simulation of an existing public office building. The format generated through the *TRNSYS17*, containing 8,760 tuples (1 line for each hour of a year) with the inputs and outputs applied in the modelling, as well as its corresponding simulation, has been respected. The proposal was to develop an experimental configuration to build an ANN model. Finally, the possible results can be compared against a reference value to substantiate the new applied methodology.

## Case study

### Modelling process and simulation

The first step carried out was the generation of synthetic data that allowed us to create the input database for our ANN model. These inputs were:

- External atmospheric conditions (dry temperature) with 8,760 tuples each year,
- Schedules for heating or cooling and ventilation with its percentage of use,
- Timetables and number of users inside the building,
- Other internal gains such as the percentage of use of lights and the number of computers running,
- The comfort conditions required in each time slot, and
- The main characteristics of the envelope, as well as the grade of infiltration of the building.

To obtain a model, the first task is to gather the maximum technical information about the building envelope to be analysed and its conditions of use, paying special attention to the possible existing internal gains, as well as its HVAC system. The basic information required to perform a correct modelling involves knowing the exact location of the building, aside from having its construction plans, to be able to define orientations and, above all, define the areas of analysis that are considered appropriate. Next, starting from the construction reports and the drawings, it is important to define the construction materials used, with their main characteristics. Once the information on the physical part of the building has been collected, it is also necessary to know the technical details of its implemented HVAC system with its operating range and limitations. The next step is to know the conditions of use of the building in question, ranging from the number of occupants in the different time slots to the characteristics of the possible equipment that can affect its internal gains, such as the type and quantity of lighting equipment, number of computers or other electronic equipment commonly used inside the building.

For our research, we have taken the Rectorate building of the University of the Basque Country, which is an office building located in the town of Leioa (Vizcaya), in Northern Spain. This building has undergone a re-construction process focused on optimizing its energy consumption. Its data collection was therefore very exhaustive, taking in the years between 2016 and 2019. It had several sensors on each floor, which made it possible to have a redundant system to guarantee that the required data concerning the internal environmental conditions and its energy consumption throughout the entire building was captured. Once all the possible information on the building had been collected, it was necessary to define the most appropriate strategy for its modelling, considering how its subsequent simulation would be developed, taking into account the objectives to be achieved and the accuracy required. Firstly, the TMY data available in the *TRNSYS17* was compared, to check if they are within of the range of the real data of the 4 years indicated. They could thus be applied, in a first phase, to define and train a first ANN model with a unique simulation based on the standard TMY. It could then be used as a reference to replicate this simulation with an ANN model. Based on all this, simplifications were considered as was the definition of more or less wide areas, according to the real measurements that were available, etc. All this would allow us to have a modelling and simulation as close as possible to reality within the existing limitations.

**Figure 1:**
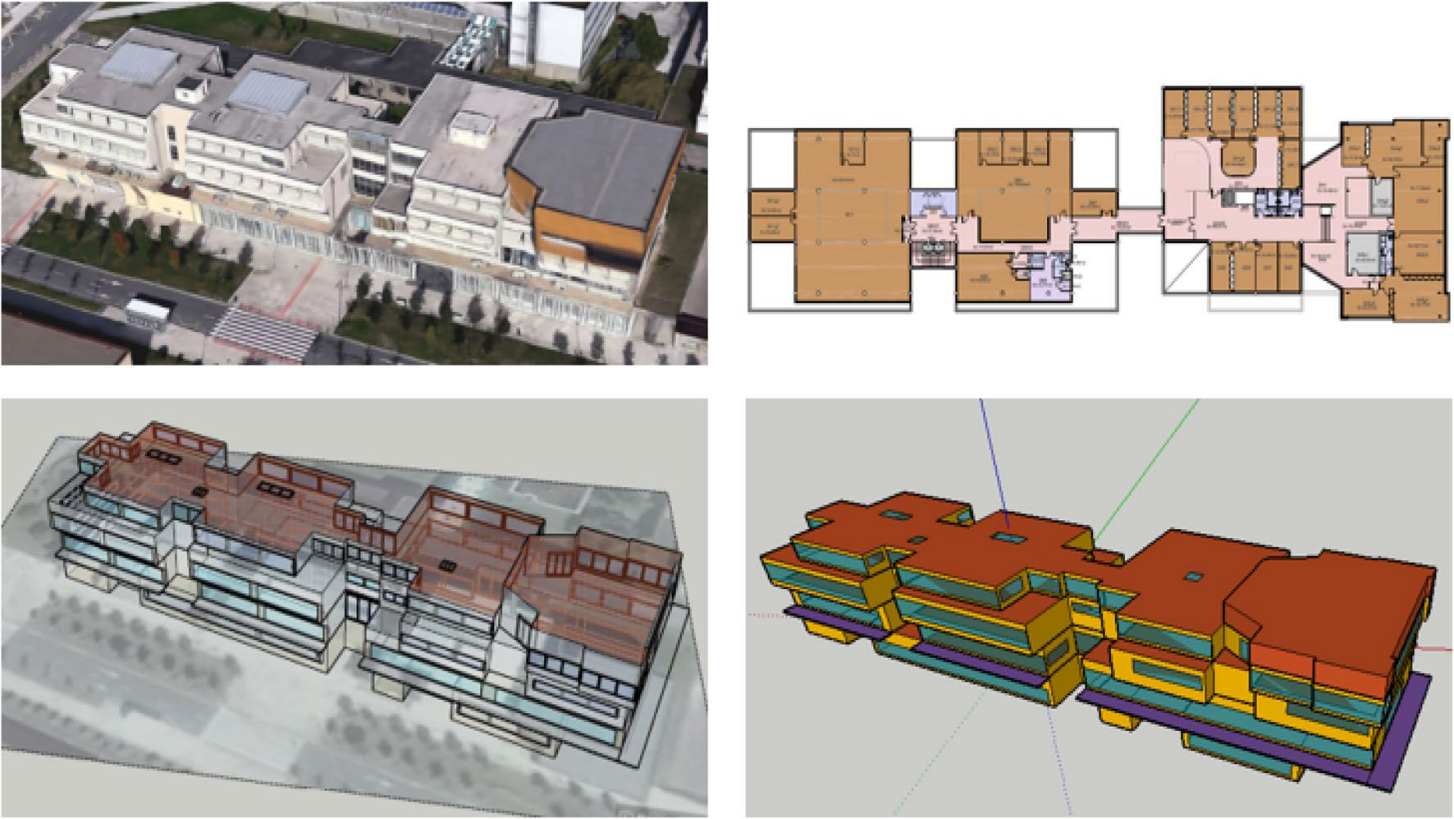
Modelling process of the University of the Basque Country Rectorate building. Photo taken by *Google Earth* and drawings developed with *SketchUp Make 2017*.

In our work, a three-dimensional model of the building under study was created using *Sketchup* software. This program has enough drawing tools to generate the required geometry of the building, with the later use of a plugin that transfers it to *TRNSYS17*. In this way, and thanks to the interaction between both software programmes, incorporating a toolbar interface in *Sketchup*, it has been possible to define the thermal zones considered, as well as windows or shadow areas. A sequence of the steps of the modelling process of the building under study is shown in Fig. 1.

After creating the geometry of the building and defining its thermal zones, windows and shadow areas with *Sketchup* and the plugin for *TRNSYS17*, an ASCII file with an IDF extension was created containing the input data that describes the building. With this modelled building geometry, the *3D-Building Wizard* of *TRNSYS17* was used to characterize its envelope, defining its walls, floors, ceilings and windows. All this is defined in the *TRNBuild*, together with all the factors that influence the thermal behaviour of a building: Enclosure, Infiltration, Heating, Refrigeration, Ventilation, Internal gains, Comfort conditions and Hours of use. Table 1 summarizes this characterization of the building under study:

**Table 1:** Relevant information and envelope's characterization of the building.

	Number of sensors	Maximum occupancy	Quantity computers	Envelope elements	Type of material	U value (W/m^2^K)	Thickness (m)
**Ground Floor**							
	**3**	**40**	**15**	Soil floor	Sand+Concrete	2.081	0.6
				Ext wall	Face Brick	2.975	0.2
				Ext roof	Slab with XPS	0.445	0.3
				Glazing Surface	Alu without TB	1.060	*4/16/4*
**First Floor**							
	**3**	**40**	**48**	Ground_floor	Slab with XPS	0.440	0.3
				Ext wall	Face Brick	2.975	0.2
				Ext_roof	Slab with XPS	0.445	0.3
				Glazing Surface	Alu without TB	1.060	*4/16/4*
**Second Floor**							
	**4**	**25**	**31**	Ground_floor	Slab with XPS	0.440	0.3
				Ext wall	Face Brick	2.975	0.2
				Ext_roof	Slab with XPS	0.445	0.3
				Glazing Surface	Alu without TB	1.060	*4/16/4*
**Third Floor**							
	**3**	**50**	**56**	Ground_floor	Slab with XPS	0.440	0.3
				Ext wall	Face Brick	2.975	0.2
				Ext_roof1	Slab without XPS	2.223	0.3
				Glazing Surface	Alu without TB	1.060	*4/16/4*

In our study, the building has been characterized defining 4 thermal zones, each of which corresponds to a floor of the building. The Simulation Study over a full year (8,760 h), obtaining data every hour through *TRNSYS17* by applying an hourly basis, was developed later. The results obtained were archived in a CSV format for further processing.

### Analysis of the data obtained

The synthetic data obtained through *TRNSYS17* and the data from real measurements taken in 2016, 2017, 2018 and 2019 in each of the thermal zones with the same CSV format have all been crosschecked. This comparison has allowed us to verify the accuracy and validity of our model. To develop this work of analysis and comparison of the data, a *Business Intelligence* application (an Extract, Transform and Load tool): *Microsoft Power BI Desktop* was used that allowed us to collect and join information from different sources: databases, spreadsheets, web services, etc., to get a data layout to make its analysis and presentation easier. This information and all the data series is shown in the link: https://app.powerbi.com/view?r=eyJrIjoiODBkNGNlM2ItNzNkNC00NTA0LTgwMDQtYTYzYzhjZDY3ZDcwIiwidCI6ImYzM2Y0ODE2LTc0OTMtNDZmYi05ZjY4LWY0NDJmNzU0ZmNhNyIsImMiOjl9.

In our case study, with 8,760 tuples for each year, the most decisive data of a building's energy systems have firstly been analysed to control the factors that could cause a significant influence, as indicated in the paper developed by Santos et al.^35^; while data that could cause errors have also been refined.

#### Site and weather conditions

**Figure 2:**
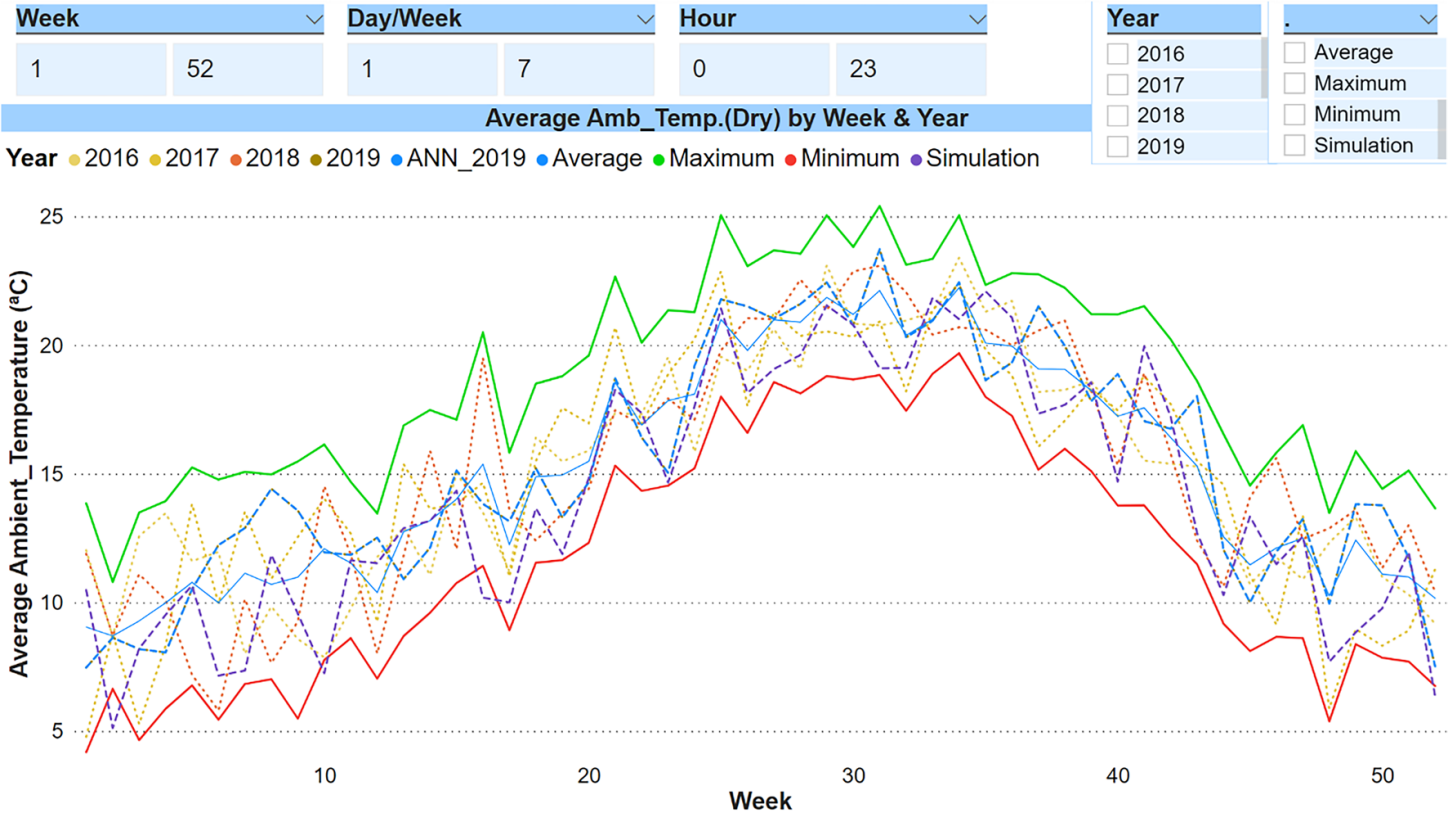
Graphs of the ambient temperatures (Dry): simulation data VS real data.

The location of the building under study determines the climatic conditions to which it is subjected, and also allows us to have a history of the data with a greater or lesser degree of precision, depending on the distance at which a meteorological station is located. In addition, it is important to clarify that the meteorological data available by default from a database of a BEPS tool are averages from several years, the so called "Typical Meteorological Year" (TMY). In our case, the data applied by default in the *TRNSYS17* application correspond to Bilbao airport (at a distance of approximately 8 km). In our case, we had the atmospheric conditions captured on-site from the building itself. Figure 2 graphically shows the outside temperature (°C) with a comparison of the data applied in the simulation versus the real data for the years from 2016 to 2019. It should be noted that, although at the annual level it might seem that there are no major differences when entering into monthly detail and above all at the hourly level, the differences between data are substantial, which means that accurate comparisons cannot be made between days of the different years; so the data must be grouped, for instance in weeks.

Considering the minimum and maximum of the real data from 2016 to 2019, it can be observed how the TMY data used in the simulation with *TRNSYS17* are within this range. However, all ambient temperature data have strong fluctuations that could lead to significant discrepancies. As the ambient temperature is a critical factor, it is very interesting to know the weather forecast, because it allows the management of the HVAC system to be anticipated in order to fulfil the comfort requirements, while also optimizing the energy consumption of the building.

#### Occupancy and operational requirements

**Figure 3:**
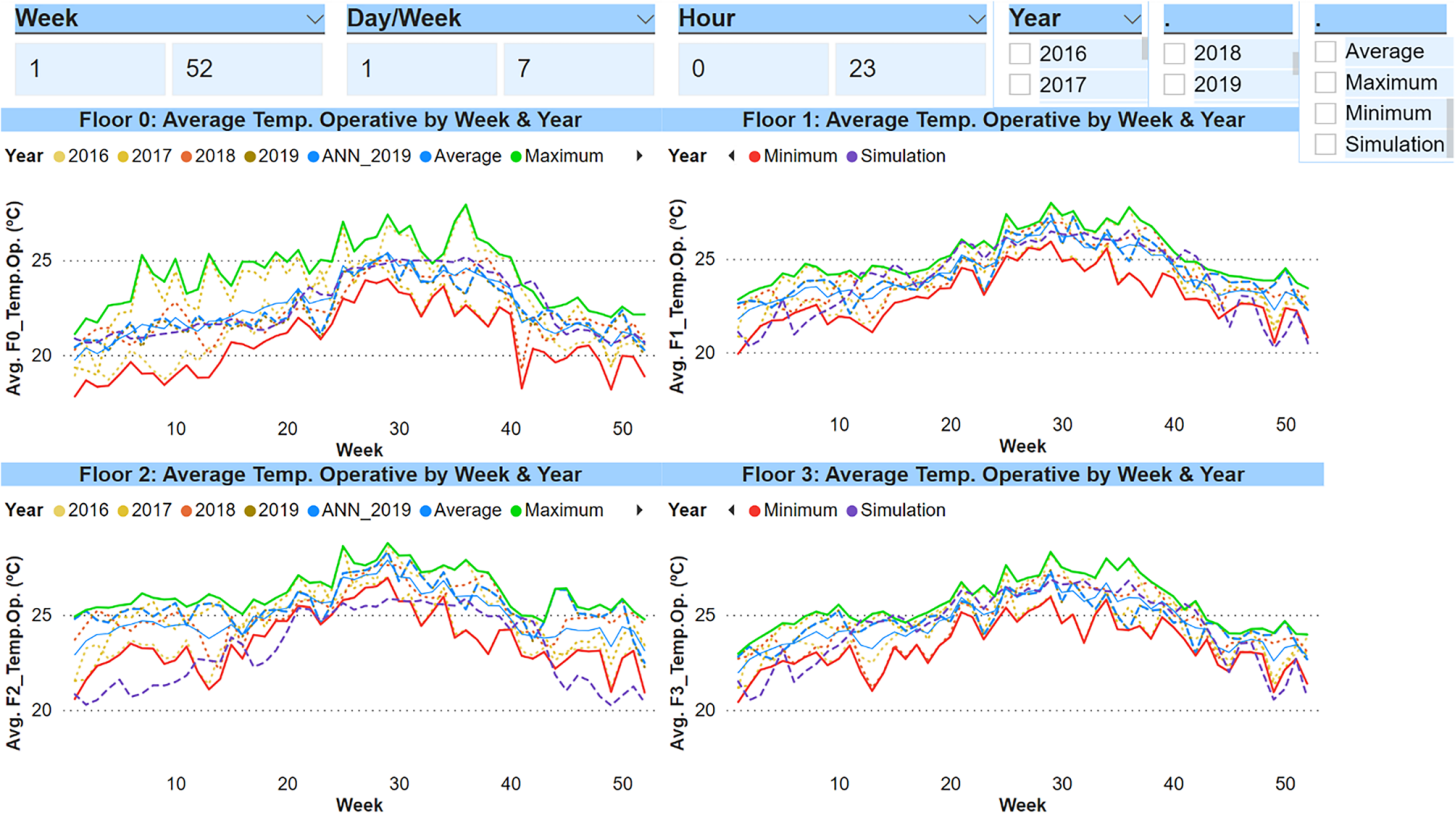
Graphs of operative temperature: simulation data VS real data.

As indicated in the Introduction section, the users of the building themselves cause important disturbances; the number of people, the computer equipment or the lights that are operating in the building have a strong influence because they generate thermal gains that affect the system. In addition, some occupants can change the comfort requirements or modify the internal environmental conditions of a thermal zone, for instance, by leaving a window open, because it changes completely the thermal behaviour of the system. Figure 3 graphically shows the operative temperature (°C) inside each defined thermal zone. As indicated in the previous section, a monthly analysis groups the information excessively and does not allow a good comparison to be established. For this reason, the information has been displayed on a weekly basis, which has also been organized to make the same week coincide in all the series in order to establish a fairer comparison between the data.

#### Building envelope

**Figure 4:**
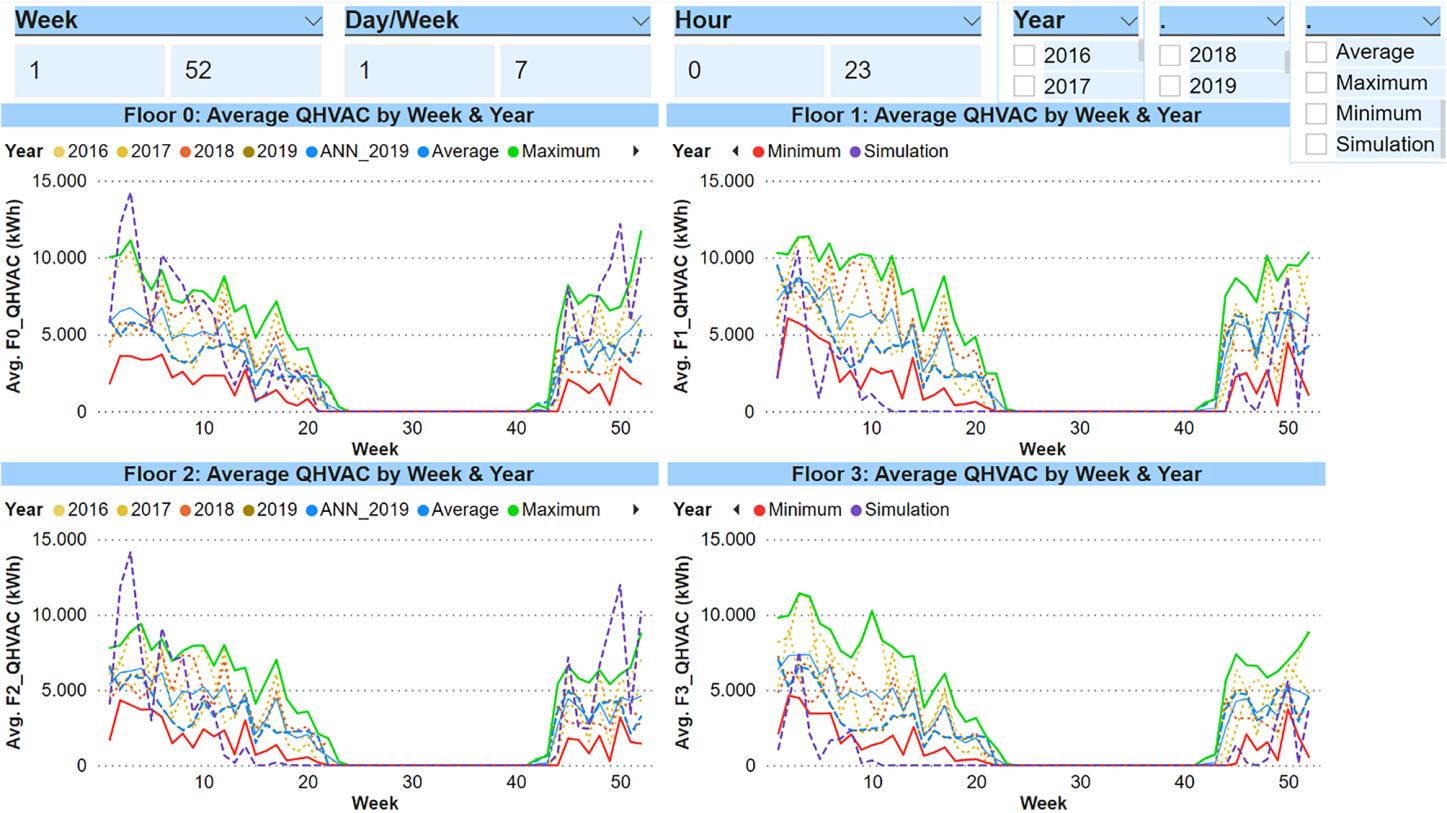
Graphs of energy consumption: simulation data VS real data.

It is not always possible to know all the information about a building envelope. Sometimes, for instance in a reconstruction of an old building, part of the information of the materials used is not available or they have simply suffered a degradation of their characteristics. In many research works, models and simulations are carried out first. After that, a calibration process is done, based on real data measured in the field. These real measurements allow adjustments to be made in the different parameters of the model so as to reach an acceptable margin of error and to consider the subsequent correct simulations. Figure 4 graphically shows the energy consumption (kWh) per floor of the simulation versus the real data. This information has been displayed weekly, for the same reasons as those explained above.

### Experimental setup to generate an ANN model

In this subsection, the process of building the artificial network based models is described. After the data gathering process described previously, they were arranged in three different subsets, in other words, the training, validation and test subsets. More specifically, the 8,760 tuples of the year 2017 were used for training the ANN, the 8,760 tuples of 2018 for validating the ANN in order to prevent overfitting, and finally, the 8,760 tuples of 2019 were taken to test the learning and the generalization capability of the trained model.

There are a number of types of ANN architectures. Among them, given the characteristics of the behaviour of the complex system to be learnt, the authors chose a specific type of ANN named Time Delayed Neural Networks (TDNN), whose characteristics are detailed by Rios et al.^37^ and Alanis et al.^38^. This type of ANNs is typically taken as networks with memory, in the sense that they can retain the dynamics of the system to be modelled. This characteristic is particularly appropriate for dealing with this problem, given the high importance of the thermal inertia underlying it. In order to acquire this feature, the TDNN networks have extra inputs in their input layer: the delayed inputs and the feedback inputs. The first are delayed versions of the input signals, while the second are the delayed versions of the signals generated by the TDNN. They are flexible in the sense that the designer can determine the number of delays of each one of these subsets of inputs.

Once the structure of the ANN has been chosen, the inputs and the outputs are determined. The outputs are the Operative Temperature and the Energy Consumption (QHVAC) that are real valued magnitudes. Regarding the inputs, the authors chose the Ambient Temperature (Dry), the occupation in persons (in function of the %Schedule by the maximum expected occupancy, as indicated in Table 1), the occupation in powered computers as it is an administrative building (in function of the % schedule by the quantity of computers, as indicated in Table 1) and the level of lights (ranged from 0 to 100% in function of the %Schedule). The %Schedule is related to the hours of use: the building only has activity during weekdays from 7:00 a.m. to 9:00 p.m., its peak activity being between 8:00 a.m. and 11:00 a.m. with 100%, varying by up to 10% in the last hour from 8:00 p.m. to 9:00 p.m.. Furthermore, the setpoint or reference for the temperature is considered as an input (with only two values: 0% meaning that the HVAC system is off and 100% when HVAC is on). As stated before, delayed versions of these signals (both inputs and outputs) are considered at the input layer of the ANNs.

For each of the two magnitudes to be predicted, different ANNs were obtained for each of the floors of the building. In order to determine the specific structure of each ANN, heuristics were used (some techniques for finding a maybe non-optimal solution, but an acceptable one in a very short time compared with a full search in the solutions space), the following being the main ones for both the Operative Temperature and the QHVAC models, as indicated in Table 2:

- Different specific ANNs were used for both Operative Temperature and Energy Consumption.
- Different specific ANNs were used for each one of the 4 different floors.
- ANNs of one hidden layer were used.
- The evaluated number of hidden nodes was not consecutive.
- There was a balance between the number of input delays and the feedback delays.
- The evaluated number of delays, for both input and feedback, was not consecutive.

**Table 2:** Definition of the ANN structure and Computational Time.

	Qty. nodes in the hidden layer	Qty. Input delays	Qty. feedback delays	Computational Time (s)
**Operative Temperature**				
Ground Floor	2	1	11	8.09
First Floor	8	13	11	12.74
Second Floor	8	5	13	13.51
Third Floor	6	3	13	10.02
**Energy Consumption**				
Ground Floor	4	13	13	32.07
First Floor	10	7	11	17.54
Second Floor	2	7	13	10.16
Third Floor	8	9	13	22.66

All the ANNs were trained using the Levenberg–Marquardt algorithm, with the Mean Squared Error "MSE" value as the metric of the achieved learning.

Finally, the time needed to train the best model for each floor is shown in the last column of Table 2, for both the Operative Temperature and the Energy Consumption. They were obtained using *Mathworks Matlab R2021b* software, and the specific characteristics of the computer used are the following: 2 *Intel Xeon* processors with a total of 28 cores, 96 GB of RAM memory and HDD type SSD, running *Microsoft Windows 10*. Having fixed the structure of the models, it is possible to retrain them again when new real data are gathered without a significant increment in the training time.

## Results

The results of the predictions made by the obtained ANN model, have been considered acceptable for establishing an effective prediction of the Operative Temperature and Energy Consumption based on the RMSE, the Mean Squared Error (MSE) and the Standard Deviation Error from each of the defined thermal zones for the Operative Temperatures and total Energy Consumption, as well as with hourly calibration criteria of the FEMP^30^ (MBE ≤  ± 10%), the IPMVP^31^ (MBE ≤  ± 5% / R^2^ > 0,75), or the ASHRAE Guideline 14^32^ (MBE ≤  ± 10% / R^2^ > 0,75). These criteria have been analysed by Fernández et al.^39^, with the aim of standardizing the calibration process of modelling a building and by Ramos et al.^40^ to clarify the typical errors that can occur in such modelling. Furthermore, the value of the calibration is very promising because the MBE values on all floors are below -3.70%, which was the reference value based on research carried out in the West block of the same building in the study^33^, as shown in Table 3:

**Table 3:** Calibration and accuracy results of the obtained ANN based model.

Year: 2019	Calibration Hourly criteria	Accuracy Results:	Value Weekly Average:	% min.	% mean	% max.
**Operative Temperature**						
**Ground Floor:**				(20.25 ºC)	(22.41 ºC)	(25.32 ºC)
**MBE (%)** *≤±5%*	**-0.45%**	RMSE (test)	0.12609 ºC	0.62%	0.56%	0.50%
**Model recommendation**		MSE (test)	0.01590 ºC^2^			
**R**^**2**^ *(> 0,75)*	**0.97**	Std. dev. error	0.01021			
**First Floor:**				(22.07 ºC)	(24.33 ºC)	(27.38 ºC)
**MBE (%)** *≤±5%*	**-0.01%**	RMSE (test)	0.01991 ºC	0.09%	0.08%	0.07%
**Model recommendation**		MSE (test)	0.00040 ºC^2^			
**R**^**2**^ *(> 0,75)*	**0.99**	Std. dev. error	0.00275			
**Second Floor:**				(22.48 ºC)	(25.56 ºC)	(28.32 ºC)
**MBE (%)** *≤±5%*	**0.02%**	RMSE (test)	0.03635 ºC	0.16%	0.14%	0.13%
**Model recommendation**		MSE (test)	0.00132 ºC^2^			
**R**^**2**^ *(> 0,75)*	**0.99**	Std. dev. error	0.00509			
**Third Floor:**				(22.67 ºC)	(24.65 ºC)	(27.32 ºC)
**MBE (%)** *≤±5%*	**-0.01%**	RMSE (test)	0.01272 ºC	0.06%	0.05%	0.05%
**Model recommendation**		MSE (test)	0.00016 ºC^2^			
**R**^**2**^ *(> 0,75)*	**0.99**	Std. dev. error	0.00174			
**Energy Consumption**						
**Ground Floor:**				(0 kWh)	(2,194 kWh)	(5,951 kWh)
**MBE (%)** *≤±5%*	**-1.23%**	RMSE (test)	120.26261 kWh	-	5.48%	2.02%
**Model recommendation**		MSE (test)	14,463.10 kWh^2^			
**R**^**2**^ *(> 0,75)*	**0.82**	Std. dev. error	16.38734			
**First Floor:**				(0 kWh)	(2,722 kWh)	(9,498 kWh)
**MBE (%)** *≤±5%*	**1.07%**	RMSE (test)	117.42150 kWh	-	4.31%	1.24%
**Model recommendation**		MSE (test)	13,787.81 kWh^2^			
**R**^**2**^ *(> 0,75)*	**0.94**	Std. dev. error	15.92995			
**Second Floor:**				(0 kWh)	(2,092 kWh)	(6,443 kWh)
**MBE (%)** *≤±5%*	**1.55%**	RMSE (test)	91.10844 kWh	-	4.35%	1.41%
**Model recommendation**		MSE (test)	8,300.75 kWh^2^			
**R**^**2**^ *(> 0,75)*	**0.93**	Std. dev. error	12.16969			
**Third Floor:**				(0 kWh)	(2,075 kWh)	(6,993 kWh)
**MBE (%)** *≤±5%*	**1.66%**	RMSE (test)	118.34496 kWh	-	5.70%	1.69%
**Model recommendation**		MSE (test)	14,005.53 kWh^2^			
**R**^**2**^ *(> 0,75)*	**0.91**	Std. Dev. Error	15.98215			

**Figure 5:**
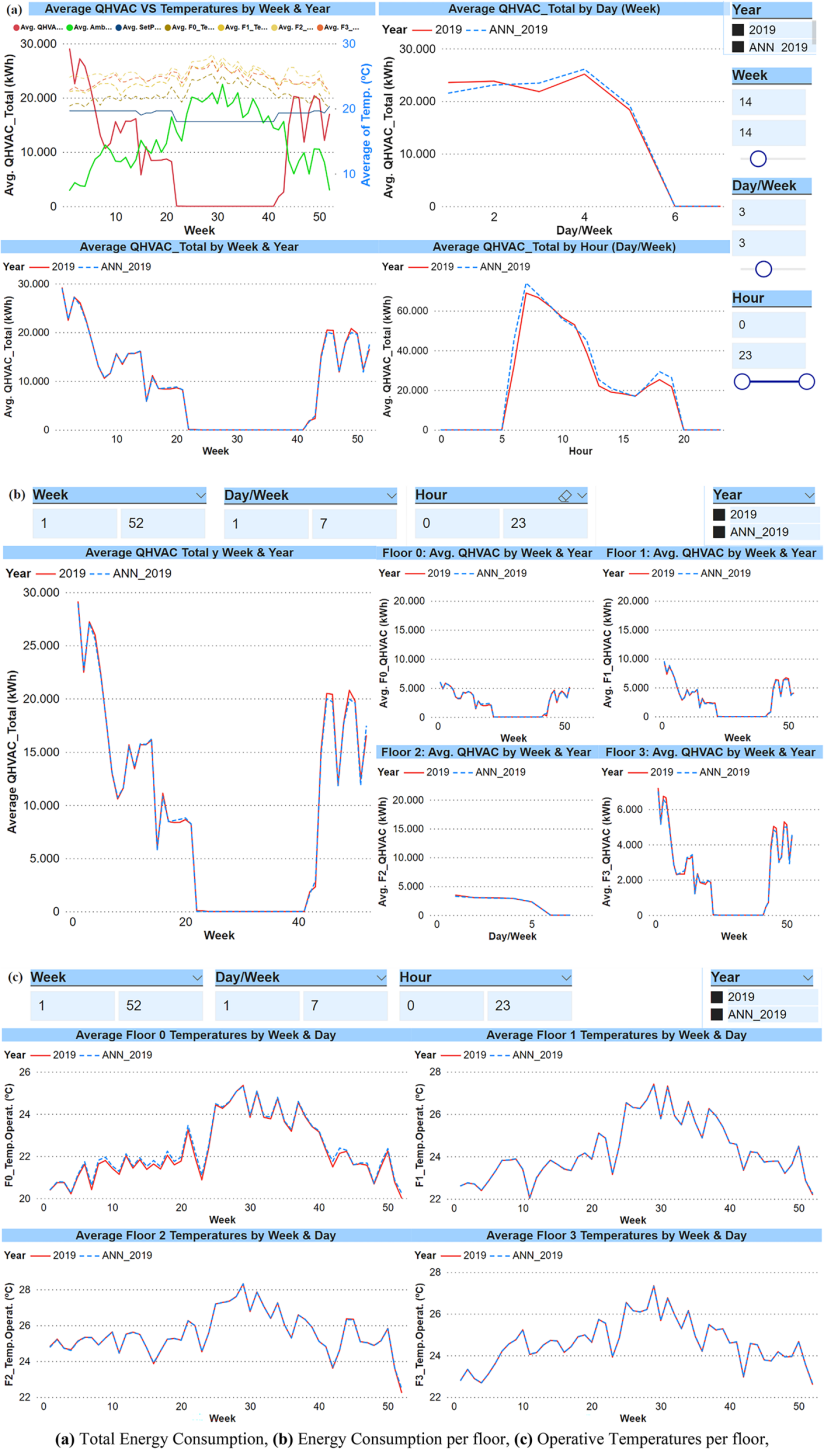
Year 2019: Comparative of Real Data VS Estimated Data by ANN. (**a**) Total Energy Consumption, (**b**) Energy Consumption per floor, (**c**) Operative Temperatures per floor.

Despite the variations that may occur in real climatic conditions, the data obtained through the ANN model are very promising. The synthetic data obtained have a very high correlation in all the comparisons made with the benchmarking carried out against the real data available. Figure 5a shows the evolution of the Real Data VS Estimated Data by the ANN of the total energy consumption (QHVAC) in kWh for the year 2019. Figure 5b shows the energy consumption in each of the defined thermal zones, i.e., each floor in this research. Even better results have been obtained in the ANN model of Operative Temperatures (°C), as shown in Fig. 5c.

The main implication of this paper is that the working hypothesis has been validated through an ANN model, where reliable estimates of the Operative Temperature and Energy Consumption of each of the floors of the public office building can be made. This approach opens up the possibility of reducing the modelling and simulation process of any building. Therefore, starting from only the real data captured from a building itself, without a modelling process and subsequent calibration; the energetic and thermal behaviour of a building can be emulated, since the ANN model itself can be used to check whether it has an acceptable correlation with the reality of the building.

## Conclusions and future challenges

This research has shown a reasonable correlation between real data for the Operative Temperature and Energy Consumption in 4 thermal zones, defined in each of the floors of a public office building, and the estimates obtained through an ANN model trained with the Levenberg–Marquardt algorithm. This research confirms that, by developing a model based on ANN, it is possible to obtain an estimate of the Operative Temperature and Energy Consumption required to air-condition each of the floors of an office building, with an acceptable degree of precision. That is, based on the results obtained, it has been concluded that it is feasible to apply an ANN model to simplify the entire modelling and simulation process described in a public office building. It should be highlighted that, in this research, the process has been simplified to demonstrate a work methodology and we have thus considered each floor of the building as a thermal zone, but this methodology is scalable. In other words, by expanding the data collection level to monitor different thermal zones, with a guarantee and the consequent increase in computational effort, it is feasible to divide a building into as many thermal zones as are considered of interest.

With this approach it is possible to simplify and reduce the cost of the building’s modelling / simulation process while optimizing its energy consumption based on forecasts of atmospheric temperatures, as well as the use of the building itself. As explained in the previous sections, the building’s modelling and simulation process is a laborious process that requires expensive technical and human resources. By developing an ANN-based model, a prediction of the Operative Temperature and Energy Consumption can be obtained with an acceptable accuracy, as can be seen by contrasting them with actual measurements from 4 consecutive years. This study shows a reasonable correlation between the real data of the Operative Temperature and Energy Consumption in the 4 thermal zones defined in each of the floors of the office building and those obtained through an algorithm generated by ANN based on the synthetic data obtained from *TRNSYS*. This implies that, with this methodology, the process of modelling and simulation of a building can be reduced, as it can be based on the real data captured from the building itself, while the calibration process can be simplified to validate the information on the thermal behaviour of any given building. This ANN model can be used to test whether it has an acceptable correlation with the reality of the building. In fact, we would not need to use *TRNSYS*, or any other modelling software, which means not having to invest time in collecting information about the building under study; and nor do we have to draw or model the building or pay for the license of a modelling program, since we can avoid all this by using the ANN to do the modelling from real data.

As future challenges, with the use of this same ANN model, a Model Predictive Control (MPC) philosophy could be applied. Based on the atmospheric forecasts that are available and using Building Automation Systems (BAS) technology, it should be possible to manage the HVAC system to optimize energy consumption based on the occupancy history and comfort requirement settings. It is even possible to improve the BAS by applying a Wireless Sensor Network (WSN) that allows occupation data and atmospheric conditions inside the building to be known in real time. The opinion of the users could also be considered, for instance, through “Smiley surveys” that the algorithm could take into account in order to make small adjustments to the environmental conditions inside of the building. In short, we consider that the ANN model developed, where all the factors that influence the thermal behaviour of a building have been modelled, as well as having the forecasts of atmospheric conditions of external temperature, allows the energy consumption of the building under control to be optimized. If the algorithm is also provided with the internal environmental conditions and the grade of occupation of the building through a WSN, this ANN-based model would have all the information necessary to optimize its HVAC system, that is, an MPC would be applied. Obviously, if the HVAC system is made up of RES facilities, reaching the nZEB standards would be totally feasible.

There is, however, one small but important detail in the case of public buildings, where there may be significant fluctuations in their grade of occupancy, and that is to include such elements as HP. These devices, with a high energy efficiency, allow us to react to these sudden variations, which can be detected by the WSN and, after the corresponding processing of the information captured in the algorithm, manage its operation. This would ensure that all the building's comfort requirements are met without penalizing its excess energy consumption.

Finally, and continuing with the application of advances achieved in the industrial sector, the strategies of the “*Industry 4.0*” should be applied in the near future to building climatization so that all their systems can be integrated and synchronized in real time. “*Industry 4.0*” considers a network model where the different processes are interconnected, instead of the traditional pyramidal model of industrial automation. This allows the creation of more flexible systems through such connections between processes and the consequent continuous exchange of their data. “*Cloud Computing*” and “*Big Data*” are fundamental for optimizing this model, together with the “*Internet of things*”, which allows data to be captured and transmitted in real time through the Internet. It also allows all the generated information to be processed and analysed through “*Cyber-Physical Systems*” made up of the appropriate hardware and algorithms. This analysis process is based on converting an immense amount of data into useful information, which allows the appropriate decisions to be taken at all times. This is what the industrial sector calls “*Business Intelligence*” and it could obviously be applied in building climatization to optimize the energy consumption, as well as its consequent reduction of CO_2_.

## Supplementary Information

Supplementary Information 1.

Supplementary Information 2.

Supplementary Information 3.

Supplementary Information 4.

Supplementary Information 5.

Supplementary Information 6.

Supplementary Information 7.

Supplementary Information 8.

Supplementary Information 9.

Supplementary Information 10.

Supplementary Information 11.

Supplementary Information 12.

## Abbreviations

ANN: Artificial neural networks
ASHRAE: American society of heating, ventilating, and air conditioning engineers
BAS: Building automation systems
BEPS: Building energy performance simulation
EPBD: Energy performance of buildings directive
FEMP: Federal energy management program
HP: Heat pump
HVAC: Heating, ventilation and air conditioning
IPMVP: International performance measurement and verification protocol
MBE: Mean bias error
MPC: Model predictive control
MSE: Mean squared error
nZEBs: Nearly-zero energy buildings
PCM: Phase change materials
PVs: Photovoltaic systems
RES: Renewable energy source
RMSE: Root mean square error
TDNN: Time delayed neural networks
TMY: Typical meteorological year
WSN: Wireless sensor network
