# Supplementary figures and images for: Energy and thermal modelling of an office building to develop an artificial neural networks model
Source: Sci Rep. 2022 May 27;12:8935. doi: 10.1038/s41598-022-12924-9 (PMC9142595; doi:10.1038/s41598-022-12924-9)

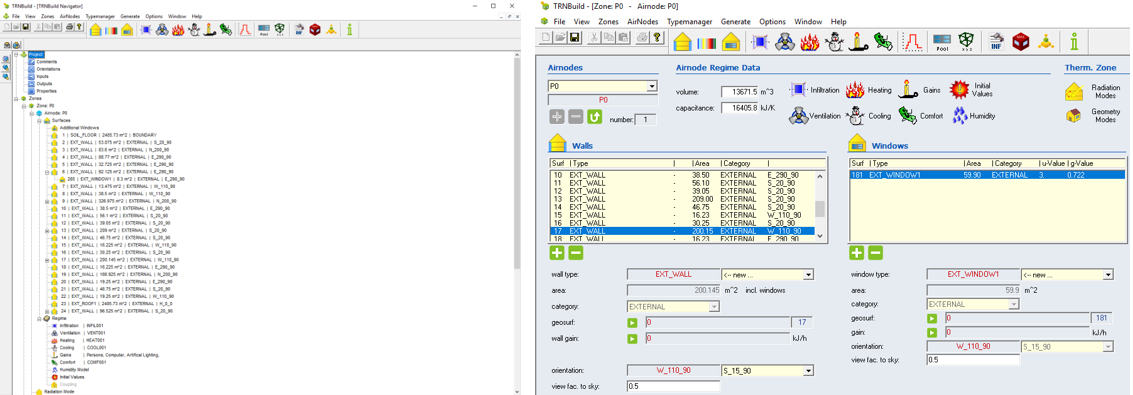

Supplement: Supplementary file 1 — Supplementary Information 1. [file 41598_2022_12924_MOESM1_ESM.png]

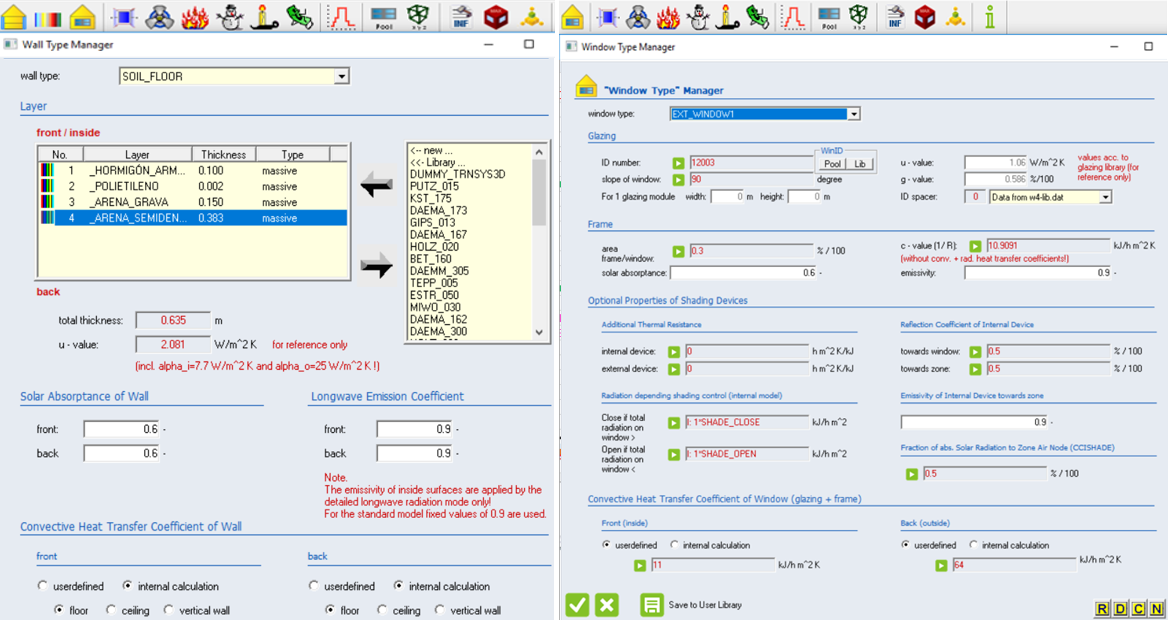

Supplement: Supplementary file 2 — Supplementary Information 2. [file 41598_2022_12924_MOESM2_ESM.png]

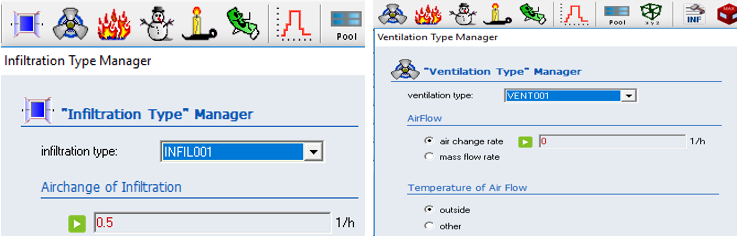

Supplement: Supplementary file 3 — Supplementary Information 3. [file 41598_2022_12924_MOESM3_ESM.png]

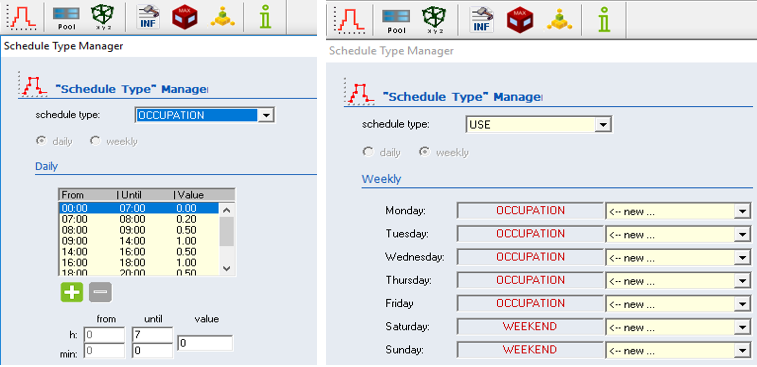

Supplement: Supplementary file 4 — Supplementary Information 4. [file 41598_2022_12924_MOESM4_ESM.png]

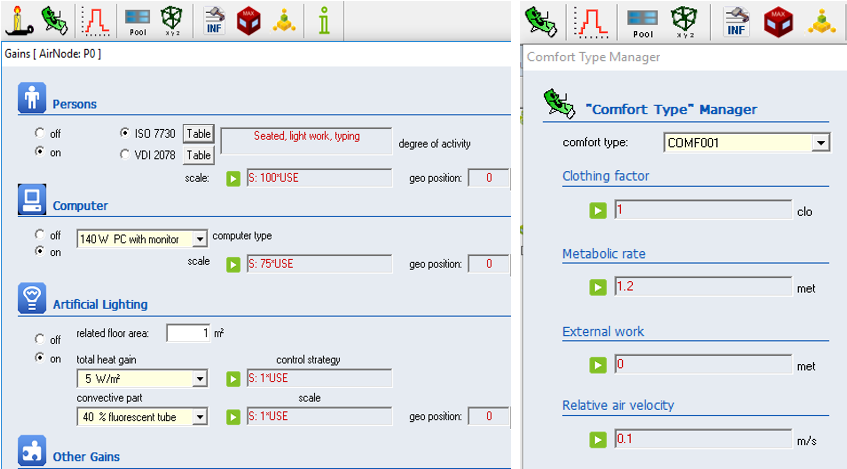

Supplement: Supplementary file 5 — Supplementary Information 5. [file 41598_2022_12924_MOESM5_ESM.png]

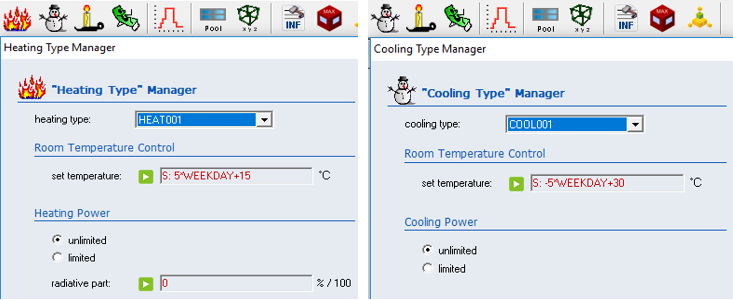

Supplement: Supplementary file 6 — Supplementary Information 6. [file 41598_2022_12924_MOESM6_ESM.png]

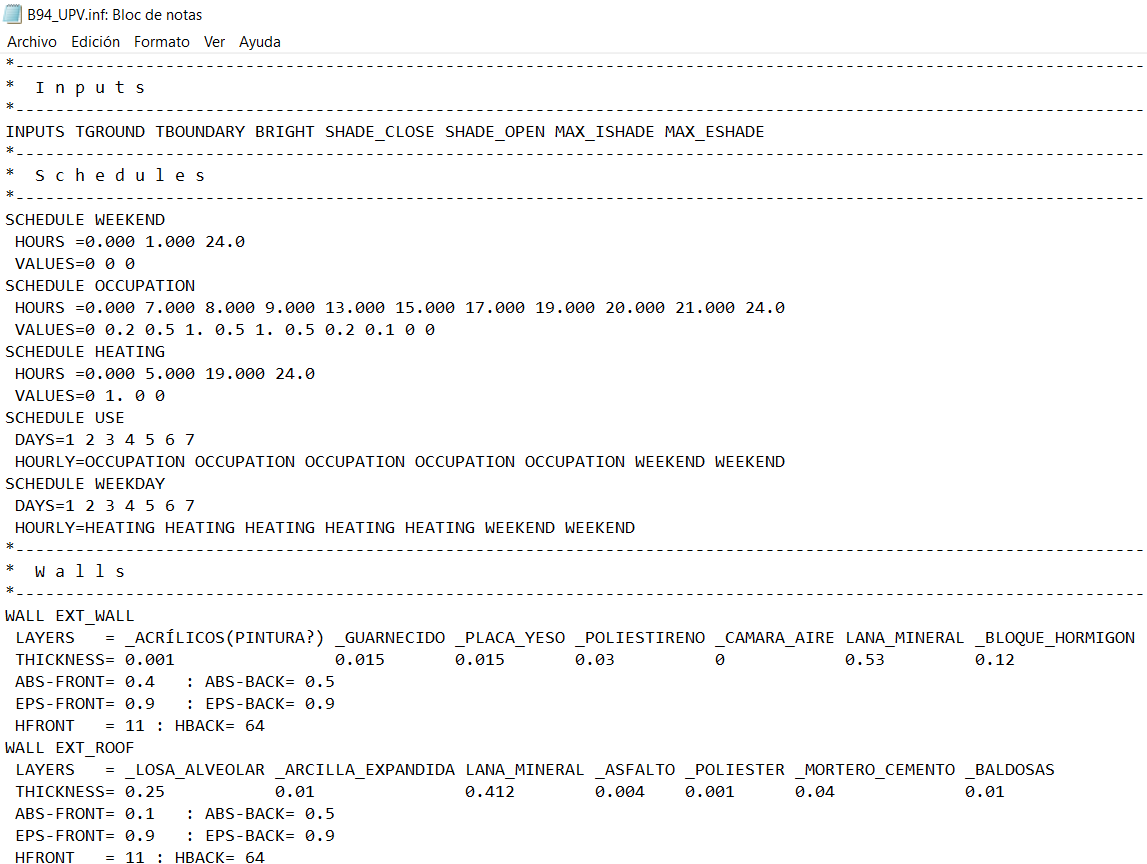

Supplement: Supplementary file 7 — Supplementary Information 7. [file 41598_2022_12924_MOESM7_ESM.png]

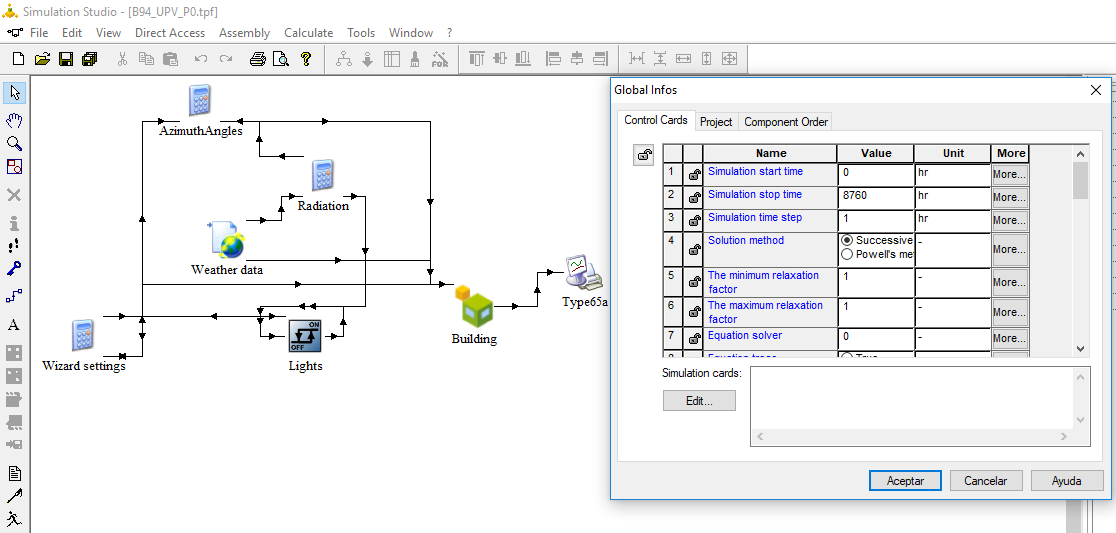

Supplement: Supplementary file 8 — Supplementary Information 8. [file 41598_2022_12924_MOESM8_ESM.png]

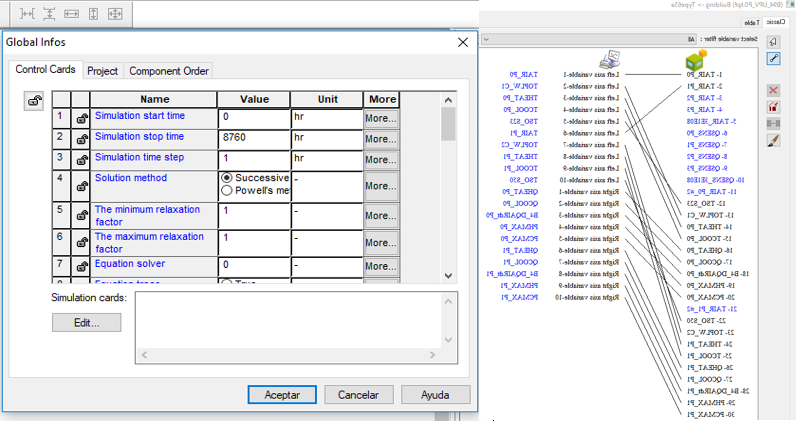

Supplement: Supplementary file 9 — Supplementary Information 9. [file 41598_2022_12924_MOESM9_ESM.png]

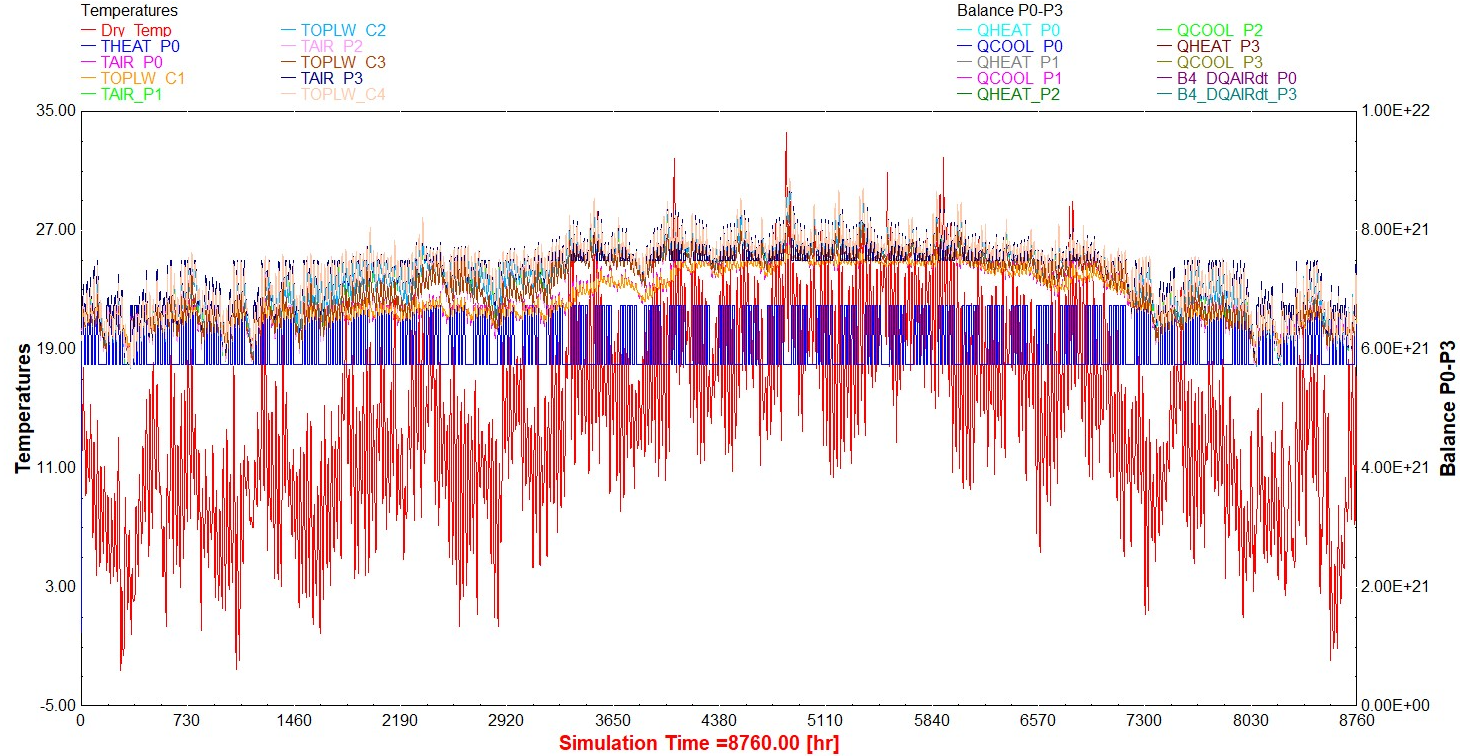

Supplement: Supplementary file 10 — Supplementary Information 10. [file 41598_2022_12924_MOESM10_ESM.png]

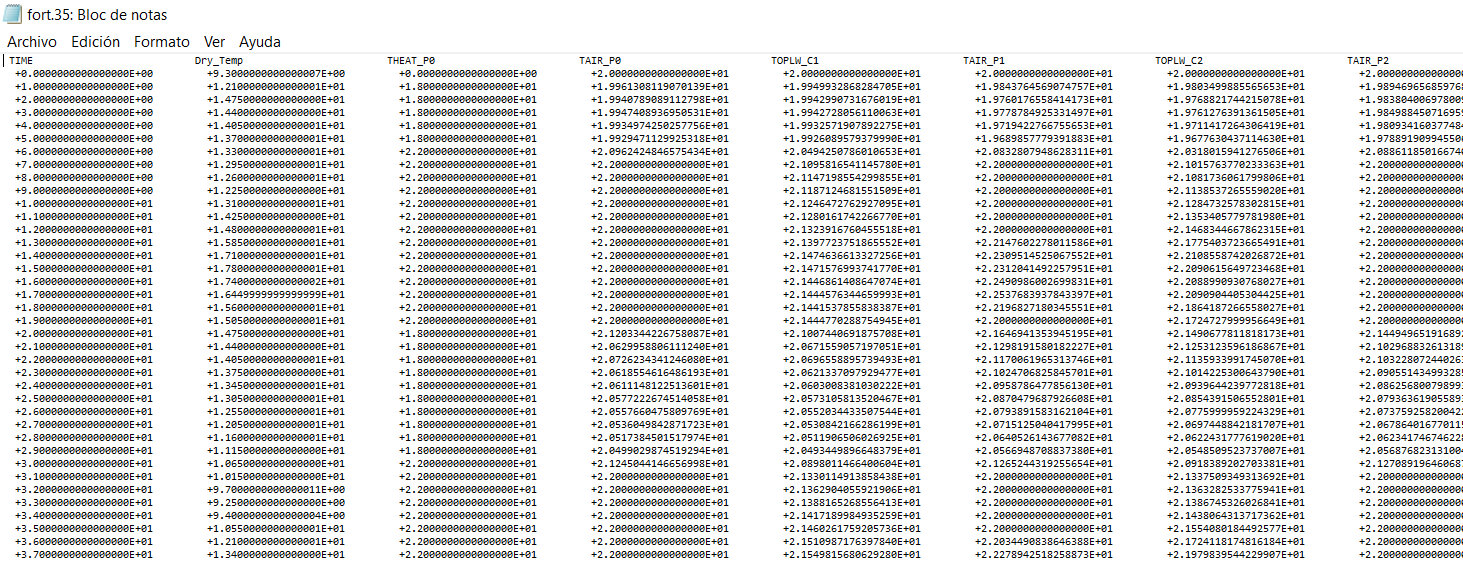

Supplement: Supplementary file 11 — Supplementary Information 11. [file 41598_2022_12924_MOESM11_ESM.png]
