# Supplementary material for: Energy and thermal modelling of an office building to develop an artificial neural networks model
Source: Sci Rep. 2022 May 27;12:8935. doi: 10.1038/s41598-022-12924-9 (PMC9142595; doi:10.1038/s41598-022-12924-9)
Supplement: Supplementary file 12 — Supplementary Information 12. [file 41598_2022_12924_MOESM12_ESM.docx]

**APPENDIX**

**Characterisation parameters of the building simulation tool**


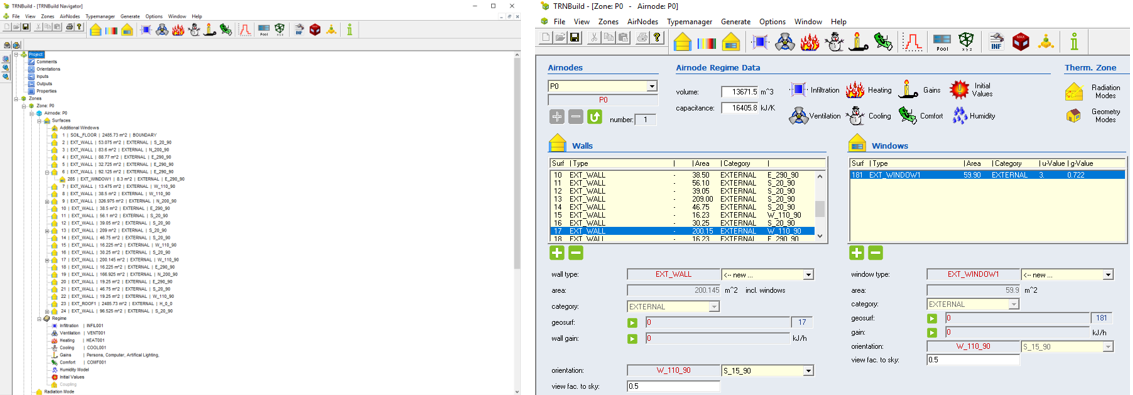


**Figure A.1.** Characterization the envelope and gains of the Rectorate building by *TRNBuild 2.0*.


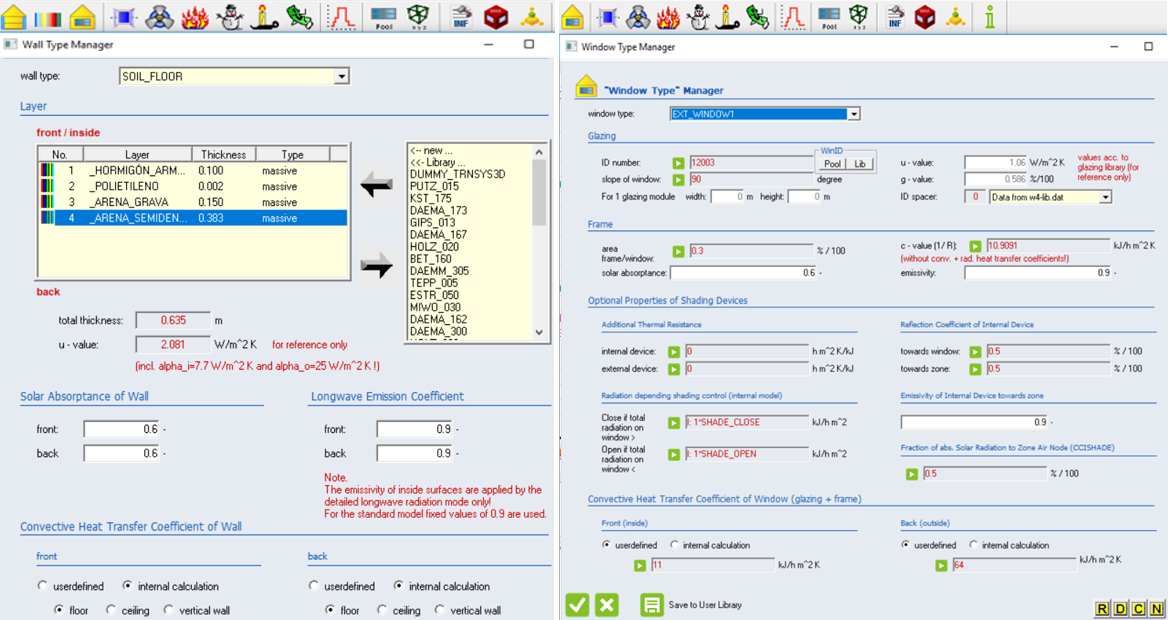


**Figure A.2.** Characterization of walls, ceilings and glazing surfaces by *TRNBuild 2.0*.


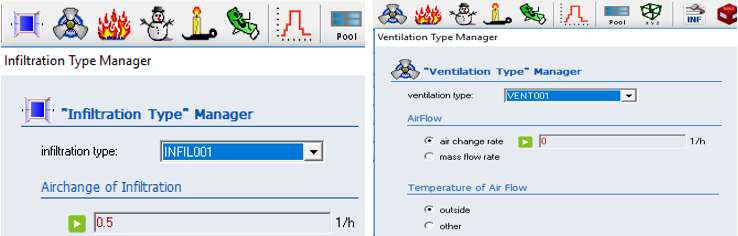


**Figure A.3.** Characterization of Infiltration and Ventilation by *TRNBuild 2.0*.


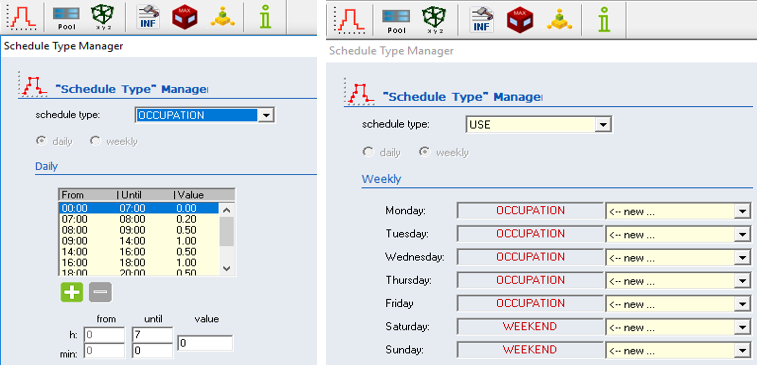


**Figure A.4.** Characterization of the Occupation and Use of the building by *TRNBuild 2.0*.


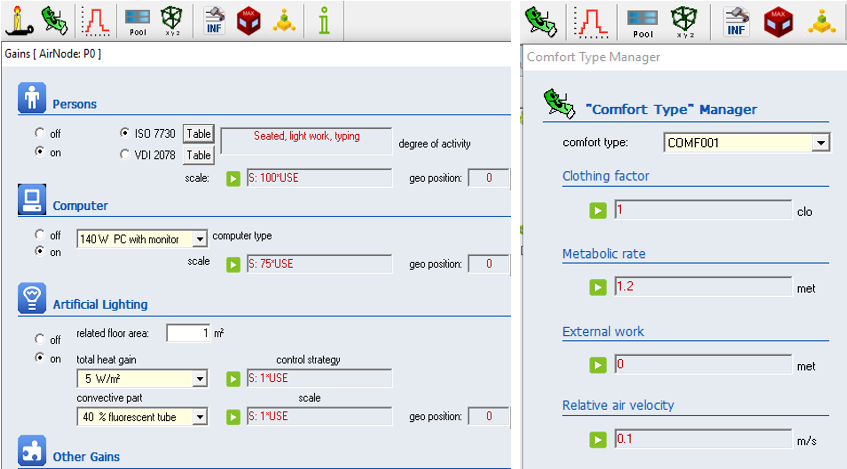


**Figure A.5.** Characterization of gains and comfort requirements by *TRNBuild 2.0*.


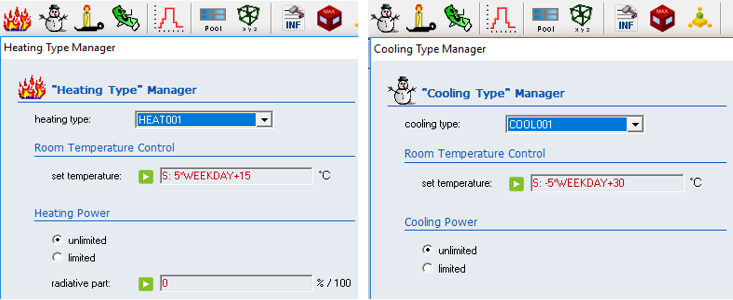


**Figure A.6.** Characterization of heating and cooling system by *TRNBuild 2.0*.


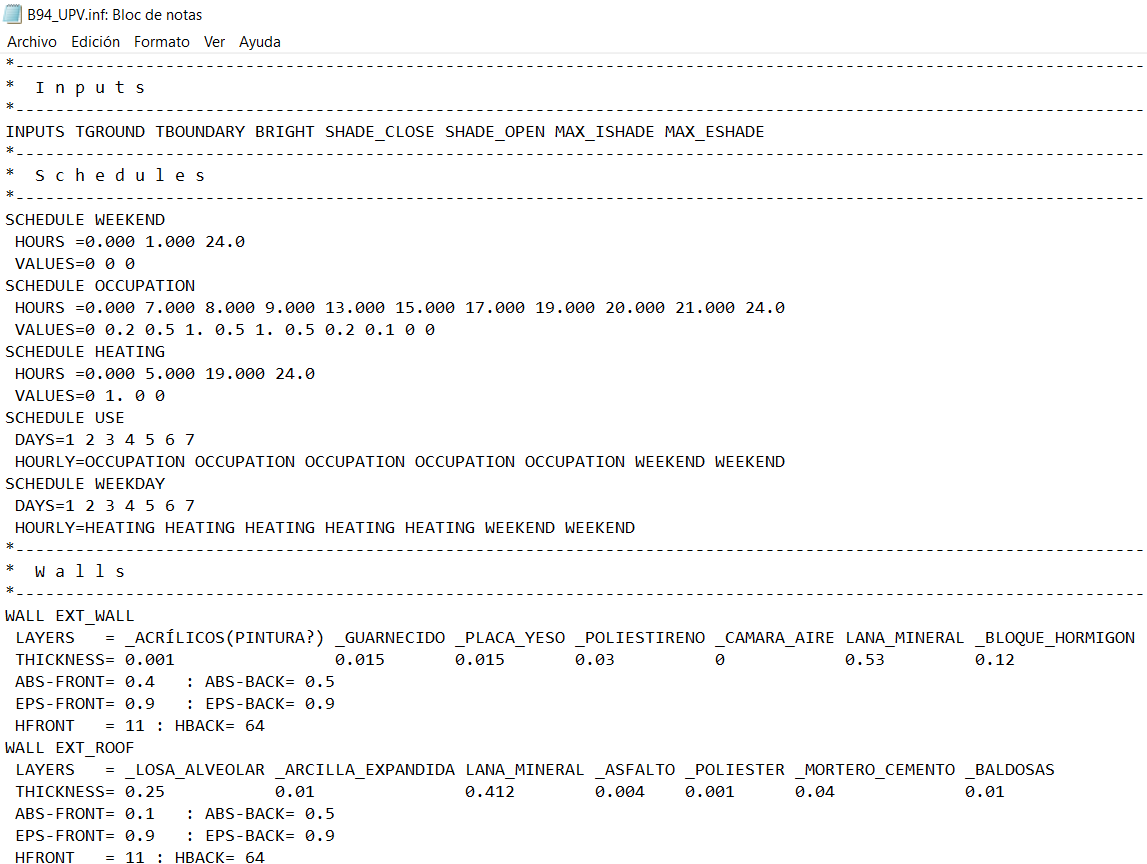


**Figure A.7.** Screenshot of the ASCII file generated after modelling of the building through *TRNBuild 2.0*.


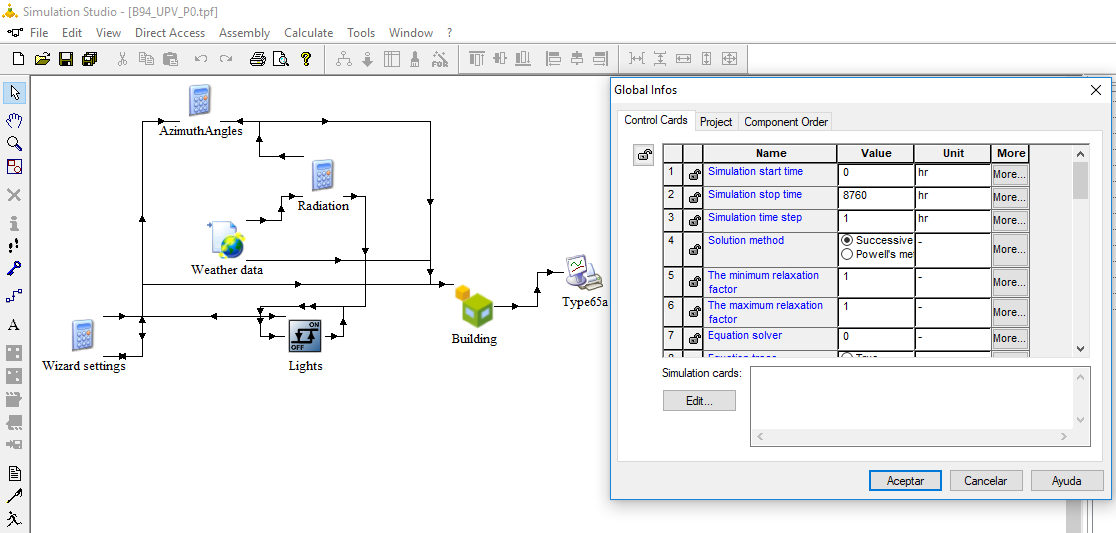


**Figure A.8.** *TRNSYS17* project defined to obtain a simulation of the building under study by *Simulation Studio*.


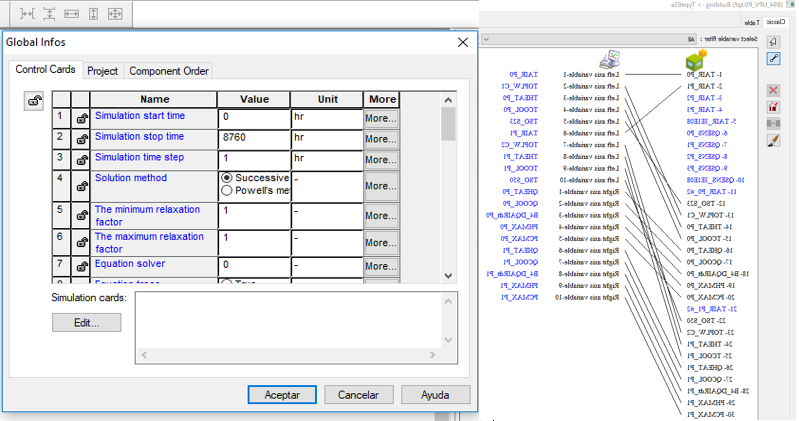


**Figure A.9.** Output connection between components *TRNSYS17*: Building–Type65a by *Simulation Studio*.

**
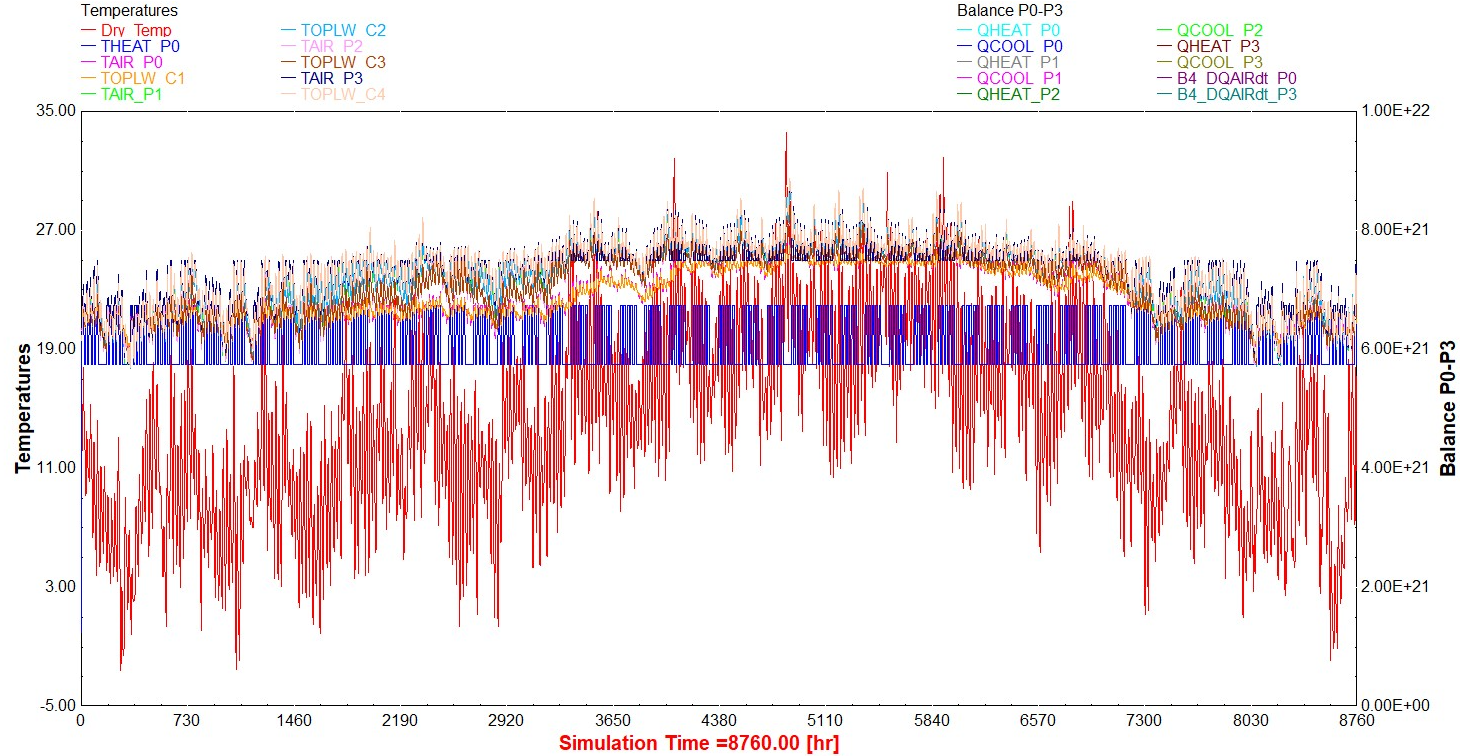
**

**Figure A.10.** Graph with the simulation results of the year obtained through *TRNSYS17* *Simulation Studio*.


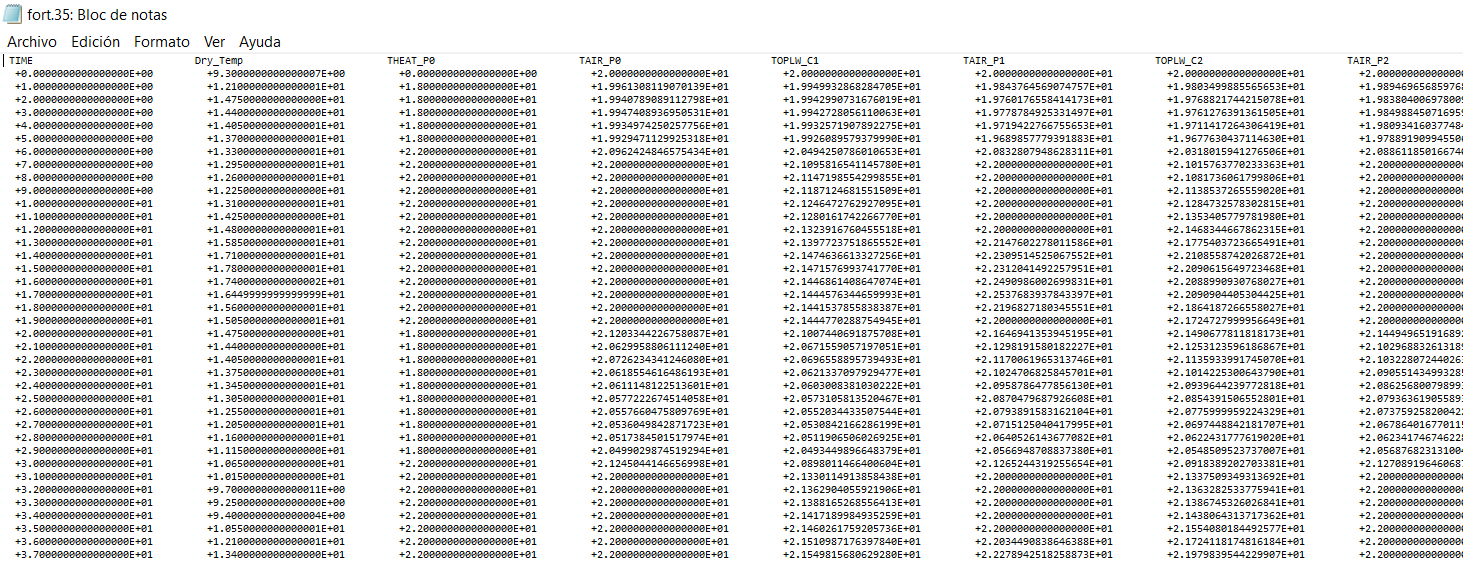


**Figure A.11.** Results of a simulation of the building in ASCII format through *TRNSYS17 Simulation Studio*.

*Note:*

The images included in this manuscript has been created using the next software:

- *SketchUp Make 2017* Version 17.2.2555 64-bit
- *TRNSYS17 Simulation Studio* Version: 5.4.0.0
- *TRNBuild 2.0* TrnsysRelease = 17.01.0028
- *Microsoft Power BI Desktop* Version: 2.103.661.0 64-bit
